# The Roles of Diffusion Kurtosis Imaging and Intravoxel Incoherent Motion Diffusion-Weighted Imaging Parameters in Preoperative Evaluation of Pathological Grades and Microvascular Invasion in Hepatocellular Carcinoma

**DOI:** 10.3389/fonc.2022.884854

**Published:** 2022-05-11

**Authors:** Fei Wang, Chun yue Yan, Cai hong Wang, Yan Yang, Dong Zhang

**Affiliations:** ^1^ Department of Medical Imaging, Luzhou People’s Hospital, Luzhou, China; ^2^ Department of Radiology, Xinqiao Hospital, Third Military Medical University, Chongqing, China; ^3^ Department of Obstetrics, Luzhou People’s Hospital, Luzhou, China

**Keywords:** hepatocellular carcinoma, microvascular invasion, grade, diffusion-weighted imaging, intravoxel incoherent motion, diffusion kurtosis imaging, meta-analysis

## Abstract

**Background:**

Currently, there are disputes about the parameters of diffusion kurtosis imaging (DKI), intravoxel incoherent motion (IVIM), and diffusion-weighted imaging (DWI) in predicting pathological grades and microvascular invasion (MVI) in hepatocellular carcinoma (HCC). The aim of our study was to investigate and compare the predictive power of DKI and IVIM-DWI parameters for preoperative evaluation of pathological grades and MVI in HCC.

**Methods:**

PubMed, Web of Science, and Embase databases were searched for relevant studies published from inception to October 2021. Review Manager 5.3 was used to summarize standardized mean differences (SMDs) of mean kurtosis (MK), mean diffusivity (MD), tissue diffusivity (D), pseudo diffusivity (D*), perfusion fraction (f), mean apparent diffusion coefficient (ADCmean), and minimum apparent diffusion coefficient (ADCmin). Stata12.0 was used to pool the sensitivity, specificity, and area under the curve (AUC). Overall, 42 up-to-standard studies with 3,807 cases of HCC were included in the meta-analysis.

**Results:**

The SMDs of ADCmean, ADCmin, and D values, but not those of D* and f values, significantly differed between well, moderately, and poorly differentiated HCC (*P* < 0.01). The sensitivity, specificity, and AUC of the MK, D, ADCmean, and ADCmin for preoperative prediction of poorly differentiated HCC were 69%/94%/0.89, 87%/80%/0.89, 82%/75%/0.86, and 83%/64%/0.81, respectively. In addition, the sensitivity, specificity, and AUC of the D and ADCmean for preoperative prediction of well-differentiated HCC were 87%/83%/0.92 and 82%/88%/0.90, respectively. The SMDs of ADCmean, ADCmin, D, MD, and MK values, but not f values, showed significant differences (*P* < 0.01) between MVI-positive (MVI+) and MVI-negative (MVI-) HCC. The sensitivity and specificity of D and ADCmean for preoperative prediction of MVI+ were 80%/80% and 74%/71%, respectively; the AUC of the D (0.87) was significantly higher than that of ADCmean (0.78) (*Z* = −2.208, *P* = 0.027). Sensitivity analysis showed that the results of the above parameters were stable and reliable, and subgroup analysis confirmed a good prediction effect.

**Conclusion:**

DKI parameters (MD and MK) and IVIM-DWI parameters (D value, ADCmean, and ADCmin) can be used as a noninvasive and simple preoperative examination method to predict the grade and MVI in HCC. Compared with ADCmean and ADCmin, MD and D values have higher diagnostic efficacy in predicting the grades of HCC, and D value has superior diagnostic efficacy to ADCmean in predicting MVI+ in HCC. However, f value cannot predict the grade or MVI in HCC.

## Introduction

Hepatocellular carcinoma (HCC) is the most common malignant tumor in the world and also one of the main causes of cancer-related death (1). Considering the specific pathogenic mechanism and epidemiological and pathological basis of the occurrence and development of HCC, early diagnosis of HCC is difficult (2). Previous studies (3, 4) have indicated that the pathological grade of HCC is closely related to patients’ prognosis; specifically, the postoperative survival rate of patients with well- and moderately differentiated HCC is significantly higher than that of patients with poorly differentiated HCC, and the 5-year postoperative recurrence rate of poorly differentiated HCC is as high as 70%. Similarly, several studies (5–7) have suggested that microvascular invasion (MVI) is an independent risk factor for recurrence and metastasis of HCC after treatment and is the most characteristic malignant biological behavior of HCC. Moreover, the postoperative recurrence rate of MVI-positive (MVI+) patients is 4.4 times higher than that of MVI-negative (MVI-) patients (8). For patients with MVI, a larger surgical resection range or ablation zone has to be employed in combination with systemic adjuvant therapy (9).

However, determination of the pathological grade and MVI of HCC mainly depends on postoperative pathological diagnosis, so there is a certain time lag. Therefore, it is extremely important to explore a noninvasive preoperative examination method to predict the pathological grade and MVI in patients with HCC. In recent years, a number of studies (10–51) have suggested that diffusion kurtosis imaging (DKI) parameters of mean kurtosis (MK) and mean diffusivity (MD) and intravoxel incoherent motion diffusion-weighted imaging (IVIM-DWI) parameters of tissue diffusivity (D), pseudo diffusivity (D*), perfusion fraction (f), mean apparent diffusion coefficient (ADCmean), and minimum apparent diffusion coefficient (ADCmin) could be used for preoperative prediction of the pathological grade or MVI in individuals with HCC. However, there are still differences and controversies as to whether these parameters can distinguish the HCC pathological grade or MVI before surgery; moreover, the preoperative prediction efficacy in previous studies was different, with large differences in each effective index and small sample size.

In 2020, a meta-analysis (52) summarized the diagnostic efficacy of ADC value (six studies, 693 HCCs) for well-differentiated HCC, and D (four studies, 304 HCCs) was better than ADC value (13 studies, 1,239 HCCs) in differentiating poorly differentiated HCC (Z = −2.718, *P* = 0.007). However, some studies (15, 25, 31, 33–35, 38, 40, 42, 49–51) were not included in that meta-analysis. Moreover, that meta-analysis did not summarize the diagnostic efficacy of IVIM-DWI parameters for MVI and did not analyze whether D*, f, MK, and MD could predict the pathological grade and MVI in individuals with HCC. In addition, it remains controversial whether D*, f, MK, and MD values could detect the HCC pathological grade or MVI before surgery (22, 25, 34, 35, 39, 46, 47).

Therefore, the aim of our meta-analysis was to comprehensively investigate whether DKI or IVIM-DWI parameters could predict the pathological grade or MVI in patients with HCC and to compare the predictive power of these parameters for the diagnosis of pathological grades and MVI+ in individuals with HCC.

## Materials and Methods

### Data Acquisition

PubMed, Web of Science, and Embase databases were searched for relevant articles published from inception to October 2021. The following search strategy was used: (a) DKI OR diffusion kurtosis imaging OR IVIM OR intravoxel incoherent motion OR DWI OR diffusion-weighted imaging OR apparent diffusion coefficient OR ADC mean value OR ADC minimum value AND hepatocellular carcinoma AND histological grade OR histopathological grade AND grading; (b) DKI OR diffusion kurtosis imaging OR IVIM OR intravoxel incoherent motion OR DWI OR diffusion weighted imaging OR apparent diffusion coefficient OR ADC mean value OR ADC minimum value AND hepatocellular carcinoma OR HCC and microvascular invasion OR microvessel invasion.

### Inclusion and Exclusion Criteria

The inclusion criteria were as follows: (a) evaluation of the diagnostic performance of DKI or IVIM or DWI for determining the presence of MVI or tumor grading in individuals with HCC using the MD and/or MK and/or D and/or D* and/or f and/or ADCmean and/or ADCmin parameters; (b) total sample not less than 20 cases; (c) available information regarding the mean/standard deviation or sensitivity/specificity of parameters for diagnosis of HCC grade or MVI; (d) the Edmondson–Steiner (ES) grade of one indicated well differentiated HCC (wdHCC), the ES grade of two indicated moderately differentiated HCC (mdHCC), and the ES grade greater than or equal to three indicated poorly differentiated HCC (pdHCC) (52). Duplicate articles, review articles, experimental animal studies, and case reports, as well as non-English publications, were excluded.

### Data Extraction

The study complied with the Preferred Reporting Items for Systematic Reviews and Meta-Analyses (PRISMA). The retrieved literature was imported into EndNote X9 (Thomas Reuters, New York, NY, USA). After removing the duplicates, FW, CYY, and CHW extracted the basic characteristics and diagnostic parameters of the included articles in strict accordance with the inclusion and exclusion criteria, and the obtained data were reviewed three times.

### Quality Assessment

The Review Manager 5.3 software (The Cochrane Collaboration, 2014) was used to evaluate the quality of the studies, referring to the Quality Assessment of Diagnostic Accuracy Studies-2 (QUADAS-2) (53). CYY and CHW independently evaluated the risk of bias and the clinical applicability of the studies in terms of patient selection, index tests, reference standards, and flow and timing. When there was a difference in opinions, the two investigators discussed the issue and reached a consensus.

### Statistical Processing

The meta-analysis was conducted using Review Manager 5.3 and Stata version 12.0 (StataCorp, College Station, TX, USA). First of all, heterogeneity was determined by means of the inconsistency index I^2^ (54, 55). A random-effects model was used when the I^2^ was above 50% or *P* was <0.05, which indicated high heterogeneity between the studies; otherwise, a fixed-effects model was applied. Second, Egger’s test or Begg’s test was used to visually and quantitatively assess the publication bias for the continuous variables, whereas Deek’s test was used to assess the publication bias of the diagnostic study. Finally, Review Manager 5.3 was used to summarize the standardized mean difference (SMD) and 95% confidence intervals (CIs) of the parameters, and Stata12.0 was used to pool the sensitivity, specificity, and area under the curve (AUC). The sensitivity analysis and subgroup analysis were used to explore the source of heterogeneity.

## Results

### Basic Characteristics of the Study

**Figure 1:**
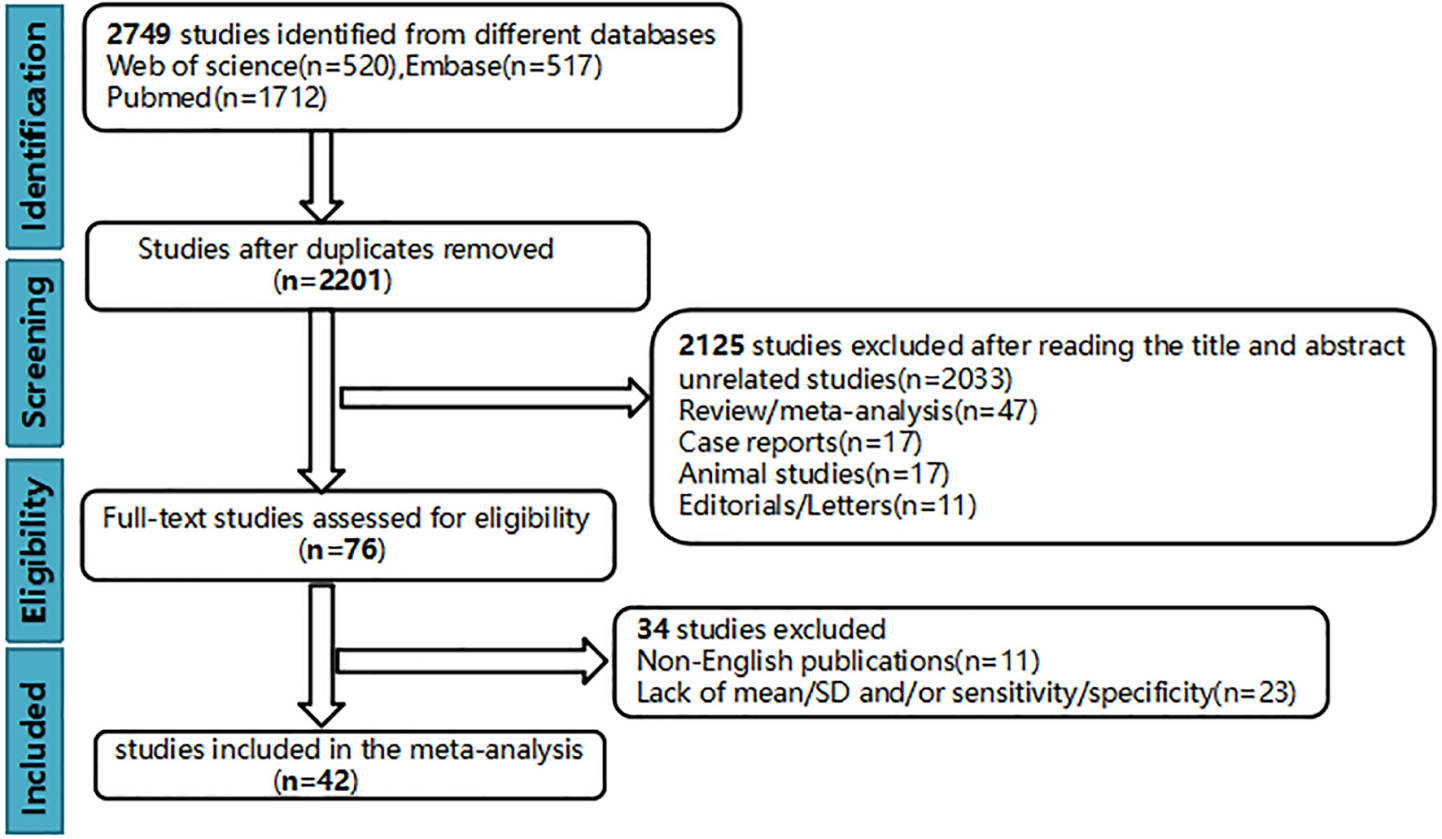
Flowchart of study selection.

Finally, 42 up-to-standard studies (10–51) with 3,807 cases of HCC were included. There were 27 studies on grading (2,172 HCCs), 11 studies on MVI (1,220 HCCs), and four studies on grading and MVI (415 HCCs). The literature screening process is shown in **Figure 1**. The basic characteristics of the included studies are shown in **Table 1**, and some parameters of diagnostic studies are shown in **Supplementary Table S1**.

**Table 1:** The basic characteristics of the studies.

Author	Published year	Country	Study design	Sample size	Research direction	Machine type	Parameters	b-values (s/mm^2^)
Muhi et al. (10)	2009	Japan	Retrospective	98	Grade	GE1.5	ADCmean	500, 1,000
Heo et al. (11)	2010	Korea	Retrospective	27	Grade	GE1.5	ADCmean	0, 1,000
Nishie et al. (12)	2011	Japan	Retrospective	52	Grade	Philips1.5	ADCmean	0, 500, 1,000
Nakanishi et al. (13)	2012	Japan	Retrospective	50	Grade	Siemens1.5	ADCmean/ADCmin	500, 1,000
Saito et al. (14)	2012	Japan	Retrospective	42	Grade	Siemens1.5	ADCmean	100, 800
Sandrasegaran et al. (15)	2013	USA	Retrospective	57	Grade	Siemens1.5	ADCmean	0, 50, 400, 500, 800
Chang et al. (16)	2014	China	Retrospective	141	Grade	GE1.5	ADCmean	0, 500
Le moigne et al. (17)	2014	France	Prospective	62	Grade	Siemens1.5	ADCmean	50, 400, 800
Woo et al. (18)	2014	Korea	Retrospective	42	Grade	Siemens3.0	ADCmean/D/D*/f	0, 25, 50, 75, 100, 200, 500, 800
Guo et al. (19)	2015	China	Prospective	27	Grade	GE3.0	ADCmean	0, 600
Tang et al. (20)	2016	China	Retrospective	74	Grade	GE3.0	ADCmean	0, 800
Iwasa et al. (21)	2016	Japan	Retrospective	42	Grade	GE1.5	ADCmean	0, 1,500
Granata et al. (22)	2016	Italy	Retrospective	62	Grade	Siemens1.5	ADCmean/D/D*/f	0, 50, 100, 200, 400, 600, 800
Shankar et al. (23)	2016	India	Prospective	20	Grade	Siemens3.0	ADCmean	0, 100, 500, 1,000
Li et al. (24)	2016	China	Retrospective	241	Grade	GE1.5	ADCmean/ADCmin	0, 800
Shan et al. (25)	2017	China	Retrospective	109	Grade	GE3.0	ADCmean/D/D*/f	0, 30, 50, 100, 150, 200, 300, 500, 800, 1,000, 1,500
Jing et al. (26)	2017	China	Retrospective	254	Grade	GE1.5	ADCmean/ADCmin	0, 600
Moriya et al. (27)	2017	Japan	Retrospective	56	Grade	Siemens1.5	ADCmin	100, 800
Ogihara et al. (28)	2018	Japan	Retrospective	42	Grade	GE1.5/3.0	ADCmean	0, 800, 1,000
Park et al. (29)	2018	Korea	Retrospective	141	Grade	Siemens1.5	ADCmean	50, 800
Zhu et al. (30)	2018	China	Retrospective	62	Grade	GE3.0	ADCmean/D/D*/f	10, 20, 40, 80, 100, 150, 200, 400, 600, 800, 1,000, 1,200
Sokmen et al. (31)	2019	Turkey	Retrospective	42	Grade	Siemens1.5	ADCmean/D	0, 50, 100, 150, 200, 300, 400, 500, 600, 700, 800, 900, 1,000, 1,100, 1,200, 1,300
Wang et al. (32)	2020	China	Retrospective	128	Grade	Siemens3.0	MD/MK/ADCmean	0, 800
Shi et al. (33)	2020	China	Prospective	52	Grade	GE3.0	D	0, 10, 20, 30, 40, 60, 80, 100, 200, 500, 800
Wu et al. (34)	2020	China	Prospective	88	Grade	GE3.0	MD/MK/ADCmean/D/D*/f	0, 20, 40, 80, 160, 200, 400, 600, 800, 1,000
Zhou et al. (35)	2021	China	Retrospective	70	Grade	GE3.0	ADCmean/D/D*/f	Unclear
Lee et al. (36)	2018	Korea	Retrospective	114	Grade/MVI	Philips3.0	ADCmean/ADCmin	0, 100, 800
Kim et al. (37)	2019	Korea	Retrospective	143	Grade/MVI	Philips3.0	ADCmean/ADCmin	0, 100, 800
Cao et al. (38)	2019	China	Retrospective	74	Grade/MVI	Siemens3.0	MD/MK/ADCmean	0, 200, 700, 1,400, 2,100
Wei et al. (39)	2019	China	Prospective	91	Grade	GE3.0	ADCmean/D/D*/f	0, 10, 20, 40, 80, 100, 150, 200, 400, 600, 800, 1,000, 1,200
Wang et al. (40)	2019	China	Retrospective	84	Grade/MVI	Siemens1.5	MD/MK/ADCmean	0, 200, 500, 1,000, 1,500, 2,000
Xu et al. (41)	2014	China	Retrospective	92	MVI	Siemens1.5	ADCmean	0, 500
Okamura et al. (42)	2016	Japan	Retrospective	75	MVI	Siemens1.5	ADCmean	0, 1,000
Huang et al. (43)	2016	China	Retrospective	51	MVI	Siemens1.5	ADCmean	0, 500
Lee et al. (44)	2017	Korea	Retrospective	197	MVI	Philips3.0	ADCmean	0, 100, 800
Zhao et al. (45)	2017	China	Retrospective	318	MVI	GE1.5	ADCmean/ADCmin	0, 800
Li et al. (46)	2018	China	Prospective	41	MVI	Philips3.0	ADCmean/D/D*/f	0, 10, 20, 40, 80, 200, 400, 600, 1,000
Zhao et al. (47)	2018	China	Retrospective	51	MVI	GE3.0	ADCmean/D/D*/f	0, 10, 20, 30, 40, 50, 60, 70, 80, 90, 100, 200, 300, 400, 500, 1,000
Chuang et al. (48)	2019	China	Retrospective	97	MVI	GE1.5	ADCmean/ADCmin	0, 400
Chen et al. (49)	2021	China	Prospective	63	MVI	uMR 770.3.0	ADCmean/D	0, 20, 40, 50, 100, 200, 500, 800, 1,500, 2,000
Wang et al. (50)	2021	China	Retrospective	100	MVI	Philips3.0/GE3.0	ADCmean	0, 100, 600
Wei et al. (51)	2019	China	Prospective	135	MVI	GE3.0	ADCmean/D/D*/f	0, 10, 20, 40, 80, 100, 150, 200, 400, 600, 800, 1,000, 1,200

ADCmean, mean apparent diffusion coefficient; ADCmin, minimum apparent diffusion coefficient; D, tissue diffusivity; D*, pseudo diffusivity; f, perfusion fraction; MVI, microvascular invasion.

### Quality Evaluation

**Figure 2:**
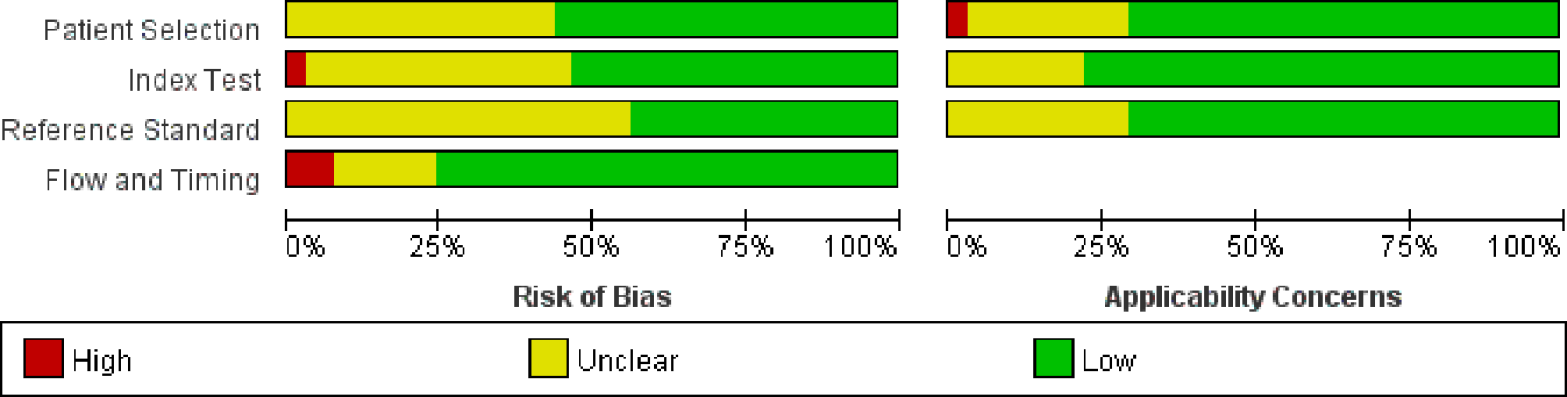
QUADAS-2 quality assessment plot.

**Figure 2** shows the quality assessment based on the QUADAS-2 scale. The overall quality of the studies was acceptable. In the patient selection domain, there was an unclear risk of bias in 18 studies because the inclusion and exclusion criteria had not been clearly reported. Eleven studies had an unclear concern, and one study had a high concern due to different inspection methods. In the index test domain, there was an unclear risk of bias in 18 studies because the information about blinding test had not been provided. Similarly, 23 studies had no information about blinding to the index test in the reference standard domain. Meanwhile, three studies had a high risk of bias in the flow and timing domain.

### Diffusion-Weighted Imaging Parameters Used for the Evaluation of Grade/Microvascular Invasion in Hepatocellular Carcinoma

#### Role of the Mean Apparent Diffusion Coefficient in the Evaluation of Grade/Microvascular Invasion in Hepatocellular Carcinoma

**Figure 3:**
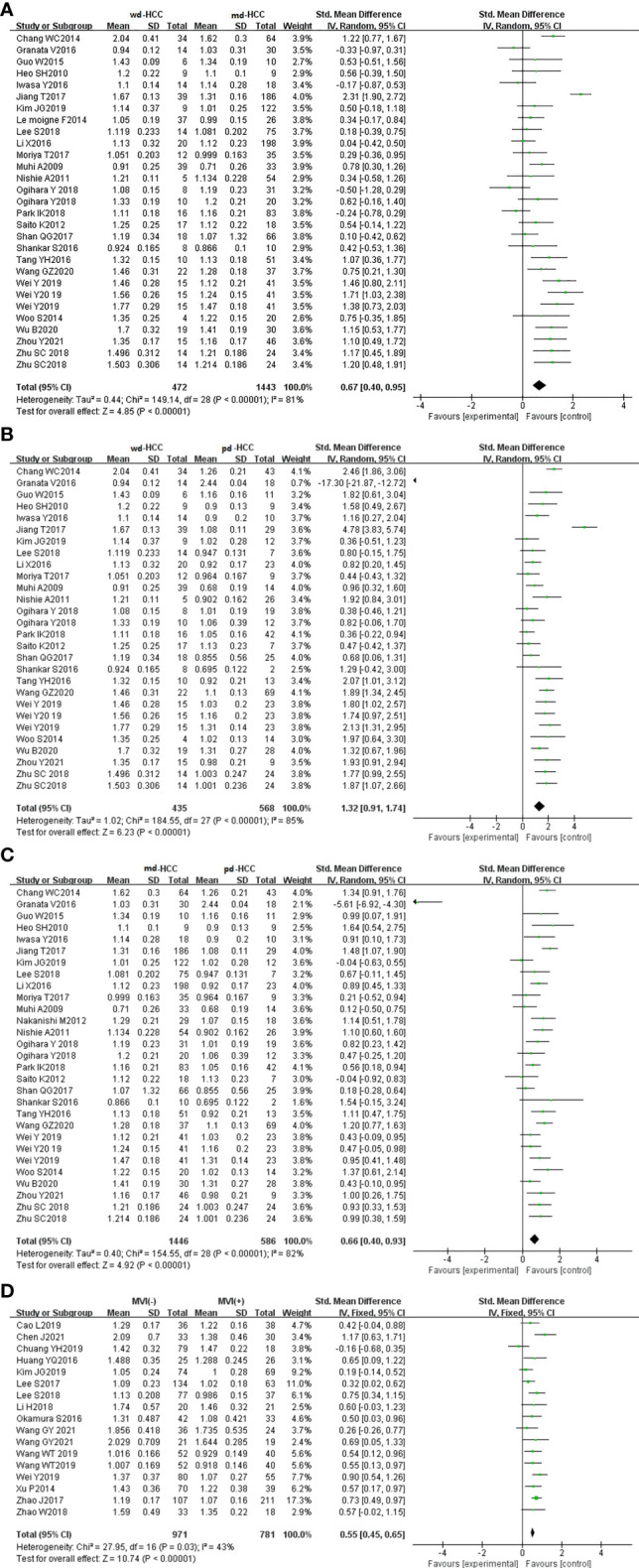
**(A)** Forest plot of ADCmean between wdHCC and mdHCC. The SMD indicated that the ADCmean of mdHCC was significantly lower than that of wdHCC. **(B)** Forest plot of the ADCmean between wdHCC and pdHCC. The SMD indicated that the ADCmean of pdHCC was significantly lower than that of wdHCC. **(C)** Forest plot of the ADCmean between mdHCC and pdHCC; the SMD indicated that the ADCmean of pdHCC was significantly lower than that of mdHCC. **(D)** Forest plot of the ADCmean between MVI- and MVI+. The SMD indicated that the ADCmean of MVI+ HCC was significantly lower than that of MVI- HCC. wd-HCC, well differentiated hepatocellular carcinoma; md-HCC, moderately differentiated hepatocellular carcinoma; pd-HCC, poorly differentiated hepatocellular carcinoma; MVI, microvascular invasion; SMD, standardized mean difference.

In 26 studies (n = 2,504), ADCmean was used to distinguish between HCC grades. There was high heterogeneity (I^2^ > 75%), so we used the random-effects model. As shown in the forest plot in **Figures 3A–C**, ADCmean positively correlated with the differentiation degree of HCC (*P* < 0.05). Egger’s test suggested no publication bias (*P* = 0.238, *P* = 0.777, *P* = 0.699). Similarly, 15 studies (n = 1,752) reported that ADCmean was used for detecting MVI. There was no significant heterogeneity (I^2^ = 45%), so the fixed-effects model was used. **Figure 3D** shows that ADCmean of MVI- HCC was significantly higher than that of MVI+ HCC (*P* < 0.01). Egger’s test suggested no publication bias (*P* = 0.958).

#### Role of the Minimum Apparent Diffusion Coefficient in the Evaluation of Grade/Microvascular Invasion in Hepatocellular Carcinoma

**Figure 4:**
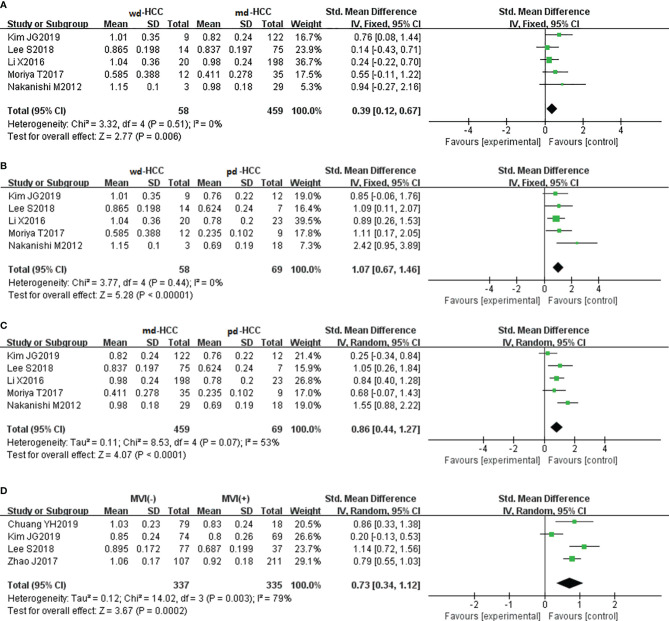
**(A)** Forest plot of the ADCmin between wdHCC and mdHCC. The SMD indicated that the ADCmin of mdHCC was significantly lower than that of wdHCC. **(B)** Forest plot of the ADCmin between wdHCC and pdHCC. The SMD indicated that the ADCmin of pdHCC was significantly lower than that of wdHCC. **(C)** Forest plot of the ADCmin between mdHCC and pdHCC. The SMD indicated that the ADCmin of pdHCC was significantly lower than that of mdHCC. **(D)** Forest plot of the ADCmin between MVI- HCC and MVI+ HCC. The SMD indicated that the ADCmin of MVI+ HCC was significantly lower than that of MVI- HCC. wd-HCC, well differentiated hepatocellular carcinoma; md-HCC, moderately differentiated hepatocellular carcinoma; pd-HCC, poorly differentiated hepatocellular carcinoma; MVI, microvascular invasion; SMD, standardized mean difference.

In five studies (n = 586), ADCmin was used for distinguishing grades. The studies (wdHCC vs. mdHCC, wdHCC vs. pdHCC) showed no significant heterogeneity (I^2^ = 0%), and the fixed-effects model was used. In contrast, the studies of mdHCC vs. pdHCC showed high heterogeneity (I^2^ = 53%), so the random-effects model was applied. As shown in **Figures 4A–C**, the ADCmin positively correlated with the differentiation degree of HCC (*P* < 0.01). Egger’s test suggested no publication bias (*P* = 0.981, *P* = 0.644, *P* = 0.614). Similarly, four studies (n = 672) reported that ADCmin was used for distinguishing MVI. These four studies had high heterogeneity (I^2^ = 79%), and the random-effects model was used. **Figure 4D** indicates that the ADCmin of MVI- HCC was significantly higher than that of MVI+ HCC (*P* < 0.01). Egger’s test suggested no publication bias (*P* = 0.699).

### Intravoxel Incoherent Motion Parameters Used for the Evaluation of Grade/Microvascular Invasion in Hepatocellular Carcinoma

#### Role of the Tissue Diffusivity Values in the Evaluation of Grade/Microvascular Invasion in Hepatocellular Carcinoma

**Figure 5:**
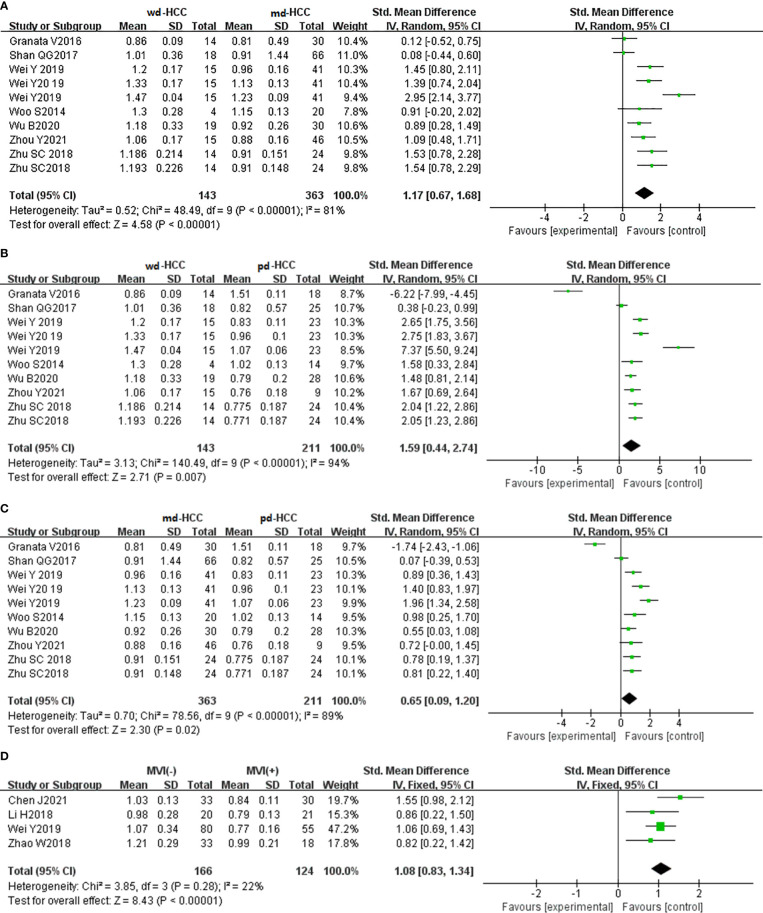
**(A)** Forest plot of the D values between wdHCC and mdHCC. The SMD indicated that the D values of mdHCC were significantly lower than those of wdHCC. **(B)** Forest plot of the D values between wdHCC and pdHCC. The SMD indicated that the D values of pdHCC were significantly lower than those of wdHCC. **(C)** Forest plot of the D values between mdHCC and pdHCC. The SMD indicated that the D values of pdHCC were significantly lower than those of mdHCC. **(D)** Forest plot of the D values between MVI- HCC and MVI+ HCC. The SMD indicated that the D values of MVI+ HCC were significantly lower than those of MVI- HCC. wd-HCC, well differentiated hepatocellular carcinoma; md-HCC, moderately differentiated hepatocellular carcinoma; pd-HCC, poorly differentiated hepatocellular carcinoma; MVI, microvascular invasion; SMD, standardized mean difference.

In seven studies (n = 711), D was used for distinguishing grades. The studies had high heterogeneity (I^2^ > 75%), and the random-effects model was used. **Figures 5A–C** show that D positively correlated with the differentiation degree of HCC (*P* < 0.05). Egger’s test (wdHCC vs. mdHCC, wdHCC vs. pdHCC) suggested no publication bias (*P* = 0.389, *P* = 0.232), and the Begg’s test of mdHCC vs. pdHCC suggested no publication bias (*P* = 0.283). Four studies (n = 672) reported that D was used for distinguishing MVI; they did not show significant heterogeneity (I^2^ = 22%), so the fixed-effects model was used. As shown in **Figure 5D**, D value of MVI- HCC was significantly higher than that of MVI+ HCC (*P* < 0.01). Egger’s test suggested no publication bias (*P* = 0.652).

#### Role of the Pseudo Diffusivity Values in the Evaluation of Grade/Microvascular Invasion in Hepatocellular Carcinoma

**Figure 6:**
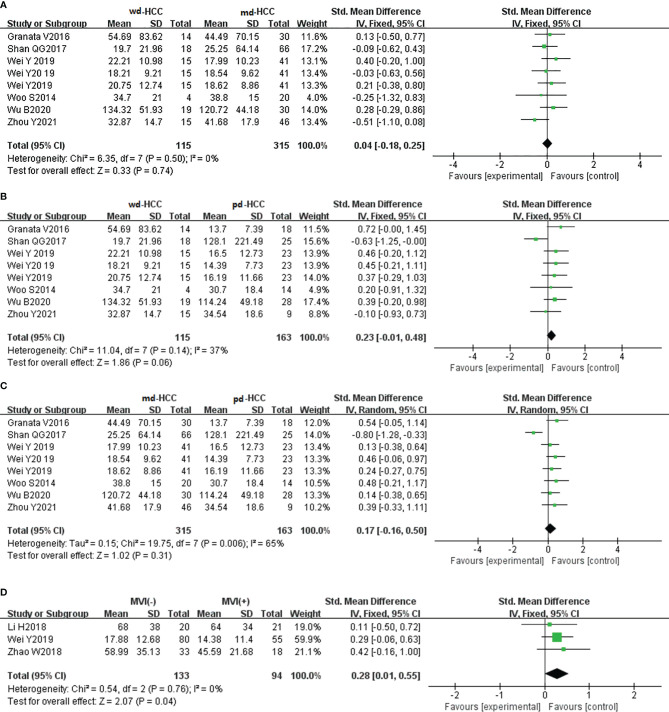
**(A–C)** Forest plot of the D* values distinguished wdHCC, mdHCC, and pdHCC. The SMDs indicated that there was no significant difference for grades in HCC. **(D)** Forest plot of the D* values between MVI- HCC and MVI+ HCC. The SMD indicated that MVI+ HCC had significantly lower D* values than MVI- HCC. wd-HCC, well differentiated hepatocellular carcinoma; md-HCC, moderately differentiated hepatocellular carcinoma; pd-HCC, poorly differentiated hepatocellular carcinoma; MVI, microvascular invasion; SMD, standardized mean difference.

In six studies (n = 593), D* was used for distinguishing grades. The studies (wdHCC vs. mdHCC, wdHCC vs. pdHCC) had no significant heterogeneity (I^2^ < 50%), so the fixed-effects model was used. The studies of mdHCC vs. pdHCC showed high heterogeneity (I^2^ = 65%), so the random-effects model was applied. As shown in **Figures 6A–C**, there was no significant difference for pathology grading in HCC (*P* > 0.05). Egger’s test suggested no publication bias (*P* = 0.510, *P* = 0.325, *P* = 0.062). Three studies (n = 227) reported that D* was used for distinguishing MVI; there was no significant heterogeneity (I^2^ = 0%), so we used the fixed-effects model. **Figure 6D** shows that D* of MVI- HCC was higher than that of MVI+ HCC (*P* < 0.05). Egger’s test suggested no publication bias (*P* = 0.560).

#### Role of the Perfusion Fraction Values in the Evaluation of Grade/Microvascular Invasion in Hepatocellular Carcinoma

**Figure 7:**
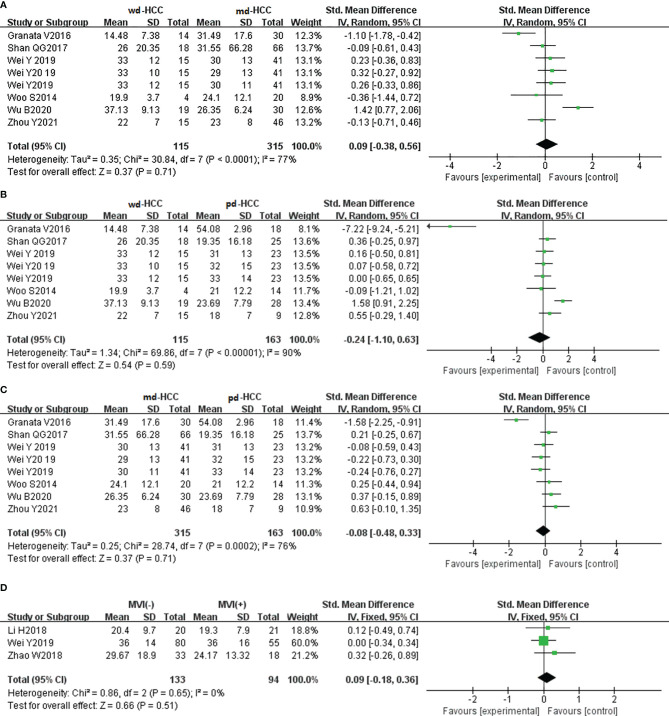
**(A–C)** Forest plot of the f values distinguished wdHCC, mdHCC, and pdHCC. The SMDs indicated that there was no significant difference for grades in HCC. **(D)** Forest plot of the f values between MVI- and MVI+. The SMD indicated that there was no significant difference for MVI in HCC. wd-HCC, well differentiated hepatocellular carcinoma; md-HCC, moderately differentiated hepatocellular carcinoma; pd-HCC, poorly differentiated hepatocellular carcinoma; MVI, microvascular invasion; SMD, standardized mean difference.

In six studies (n = 593), f was used for distinguishing grades. The studies had high heterogeneity (I^2^ > 75%), so we used the random-effects model. As shown in **Figures 7A–C**, there was no significant difference for pathology grading in HCC (*P* > 0.05). Egger’s test suggested no publication bias (*P* = 0.713, *P* = 0.100, *P* = 0.967). Three studies (n = 227) reported that f was used for distinguishing MVI. They had no significant heterogeneity (I^2^ = 0%), so the fixed-effects model was used. As shown in **Figure 7D**, f did not distinguish MVI+ HCC from MVI- HCC (*P* > 0.05). Begg’s test suggested no publication bias (*P* = 0.999).

### Diffusion Kurtosis Imaging Parameters Used for the Evaluation of Grade/Microvascular Invasion in Hepatocellular Carcinoma

#### Role of the Mean Diffusivity Values in the Evaluation of Grade/Microvascular Invasion in Hepatocellular Carcinoma

**Figure 8:**
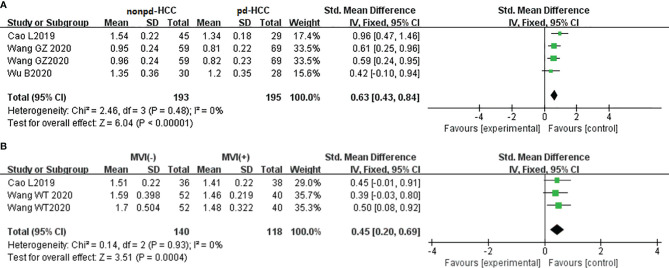
**(A)** Forest plot of the MD values between non-pdHCC and pdHCC. The SMD indicated significantly lower MD values in pdHCC than those in non-pdHCC. **(B)** Forest plot of MD values between MVI- and MVI+. The SMD indicated significantly lower MD values in MVI+ HCC than those in MVI- HCC. pd-HCC, poorly differentiated hepatocellular carcinoma; MVI, microvascular invasion; SMD, standardized mean difference.

In three studies (n = 388), MD was used for distinguishing grades. There was no significant heterogeneity (I^2^ = 0%), so we used the fixed-effects model. **Figure 8A** shows that the MD value of pdHCC was significantly lower than that of non-pdHCC (*P* < 0.01). Egger’s test suggested no publication bias (*P* = 0.582). Two studies (n = 258) reported that MD was used for distinguishing MVI; they did not show significant heterogeneity (I^2^ = 0%), and the fixed-effects model was used. **Figure 8B** shows that the MD of MVI- HCC was significantly higher than that of MVI+ HCC (*P* < 0.01). Egger’s test suggested no publication bias (*P* = 0.870).

#### Role of the Mean Kurtosis Values in the Evaluation of Grade/Microvascular Invasion in Hepatocellular Carcinoma

**Figure 9:**
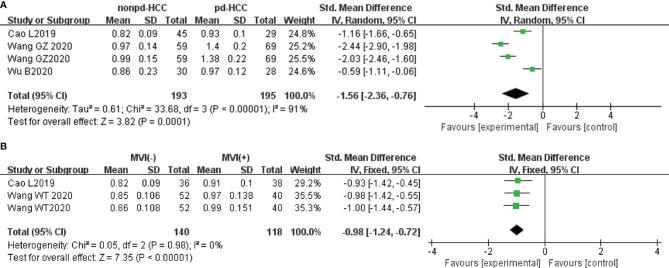
**(A)** Forest plot of the MK values between non-pdHCC and pdHCC. The SMD indicated significantly higher MK values in pdHCC than those in non-pdHCC. **(B)** Forest plot of MK values between MVI- and MVI+. The SMD indicated significantly higher MK values in MVI+ HCC than those in MVI- HCC. pd-HCC, poorly differentiated hepatocellular carcinoma; MVI, microvascular invasion; SMD, standardized mean difference.

In three studies (n = 388), the MK was used for distinguishing grades. There was highly significant heterogeneity (I^2^ > 75%), so we used the random-effects model. **Figure 9A** shows that the MK value of non-pdHCC was significantly lower than that of pdHCC (*P* < 0.01). Begg’s test suggested no publication bias (*P* = 0.308). Two studies (n = 258) reported that the MK was used to distinguish MVI. These studies did not show significant heterogeneity (I^2^ = 0%), so the fixed-effects model was used. **Figure 9B** shows that the MK of MVI- HCC was significantly lower than that of MVI+ HCC (*P* < 0.01). Egger’s test suggested no publication bias (*P* = 0.179).

### Sensitivity Analysis

#### Sensitivity Analysis of the Parameters for Distinguishing Microvascular Invasion in Hepatocellular Carcinoma

First, the SMDs of each parameter for distinguishing MVI changed little after the combination of transformation random-effects model and fixed-effects model. Moreover, after excluding each study one by one, the results of the sensitivity analysis (**Supplementary Figures S1A–G**) suggested that the studies of ADCmean, D value, D* value, f value, MD value, and MK value, but not ADCmin value, were stable and reliable to identify MVI- HCC vs. MVI+ HCC. After removing the study by Kim et al. (37), the result of ADCmin in discriminating MVI- vs. MVI+ HCC was stable and reliable (SMD = 0.87, *P* < 0.00001, **Supplementary Figure S2**). The I^2^ decreased from 79% to 1%, which suggested that the excluded study was likely the source of heterogeneity.

#### Sensitivity Analysis of the Parameters for Distinguishing Grades in Hepatocellular Carcinoma

After excluding each study one by one, the results of the sensitivity analysis (**Supplementary Figures S3A–C–S7A–C**) suggested that the other studies were stable and reliable, except for the ADCmean value to identify wdHCC vs. mdHCC, and the D value to identify wdHCC vs. pdHCC and mdHCC vs. pdHCC. After removing the study by Jiang et al. (26), the result of the ADCmean in discriminating wdHCC vs. mdHCC was stable and reliable (SMD = 0.61, *P* < 0.00001, **Supplementary Figure S8**). After removing the studies by Shan et al. (25) and Granata et al. (22), the results of the D values in discriminating the D values in discriminating wdHCC vs. pdHCC and were stable and reliable (SMD = 2.48, SMD = 1.01, *P* < 0.00001; **Supplementary Figures S9, S10**). The heterogeneity was lower than before, which suggested that these studies were likely the source of heterogeneity.

### Diagnostic Performance

**Figure 10:**
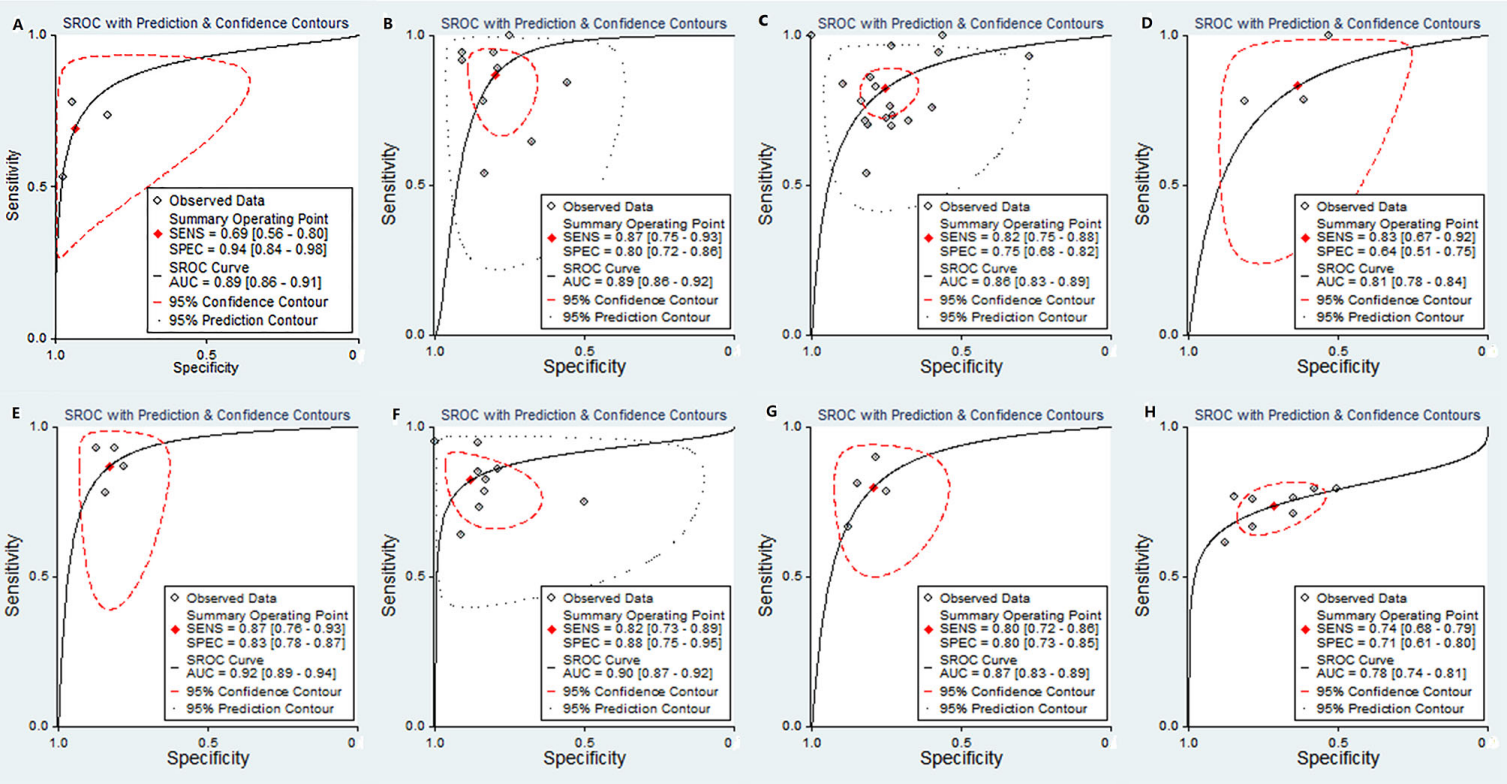
SROC plots of the MK value **(A)**, D value **(B)**, ADCmean **(C)**, and ADCmin **(D)** for discriminating pdHCC. SROC plots of the D value **(E)** and ADC mean **(F)** for discriminating wdHCC. SROC plots of the D value **(G)** and ADC mean **(H)** for discriminating MVI+ in HCC. SROC, summary receiver operating characteristic; AUC, area under curve; pdHCC, poorly differentiated hepatocellular carcinoma; wdHCC, well differentiated hepatocellular carcinoma; MVI, microvascular invasion; HCC, hepatocellular carcinoma.

The pooled sensitivity, specificity, positive likelihood ratio (PLR), negative likelihood ratio (NLR), diagnostic odds ratio (DOR), and the AUCs of the parameters are listed in **Table 2**. The AUCs of the MK, D value, ADCmean, and ADCmin for preoperative prediction of pdHCC were 0.89 (95% CI: 0.86–0.91), 0.89 (95% CI: 0.86–0.92), 0.86 (95% CI: 0.83–0.89), and 0.81 (95% CI: 0.78–0.84), respectively, as shown in **Figures 10A–D**. Deek’s test suggested no publication bias (*P* = 0.298, *P* = 0.473, *P* = 0.684, *P* = 0.093). Similarly, the AUCs of the D and ADCmean for preoperative prediction of wdHCC were 0.92 (95% CI: 0.89–0.94) and 0.90 (95% CI: 0.87–0.92), respectively, as shown in **Figures 10E, F**. Deek’s test suggested no publication bias (*P* = 0.178, *P* = 0.066). Furthermore, the AUCs of the D and ADCmean for preoperative prediction of MVI+ HCC were 0.87 (95% CI: 0.83–0.89) and 0.78 (95% CI: 0.74–0.81), respectively (*Z* = −2.208, *P* = 0.027; **Figures 10G, H** **)**. Deek’s test suggested no publication bias in terms of D (*P* = 0.331), but there was a certain publication bias regarding ADCmean (*P* = 0.024).

**Table 2:** The diagnostic performance assessed by the parameters.

Indicators	AUC (95% CI)	Sensitivity (95% CI)	Specificity (95% CI)	PLR (95% CI)	NLR (95% CI)	DOR (95% CI)
**Poorly differentiated HCC**						
MK	0.89 (0.86, 0.91)	0.69 (0.56, 0.80)#	0.94 (0.84, 0.98)&	10.7 (4.4, 26.0)	0.33 (0.23, 0.48)	32 (13, 80)
D	0.89 (0.86, 0.92)	0.87 (0.75, 0.93)#	0.80 (0.72, 0.86)#	4.4 (2.9, 6.5)	0.17 (0.08, 0.33)	26 (10, 68)
ADCmean	0.86 (0.83, 0.89)	0.82 (0.75, 0.88)#	0.75 (0.68, 0.82)#	3.4 (2.5, 4.5)	0.23 (0.17, 0.33)	14 (8, 24)
ADCmin	0.81 (0.78, 0.84)	0.83 (0.67, 0.92)&	0.64 (0.51, 0.75)#	2.3 (1.7, 3.1)	0.27 (0.13, 0.52)	9 (4, 20)
**Well-differentiated HCC**						
ADCmean	0.90 (0.87, 0.92)	0.82 (0.73, 0.89)#	0.88 (0.75, 0.95)#	7.0 (3.0, 16.2)	0.20 (0.12, 0.34)	34 (10, 120)
D	0.92 (0.89, 0.94)	0.87 (0.76, 0.93)&	0.83 (0.78, 0.87)&	5.1 (3.8, 6.9)	0.16 (0.08, 0.30)	32 (14, 73)
**MVI(+) vs. MVI(-)**						
ADCmean	0.78 (0.74, 0.81)	0.74 (0.68, 0.79)&	0.71 (0.61, 0.80)#	2.6 (1.9, 3.5)	0.37 (0.30, 0.45)	7 (5, 11)
D	0.87 (0.83, 0.89)	0.80 (0.72, 0.86)&	0.80 (0.73, 0.85)&	3.9 (2.9, 5.3)	0.25 (0.18, 0.36)	15 (9, 27)

&, the fixed effect model; #, the random effect model; ADCmean, mean apparent diffusion coefficient; ADCmin, minimum apparent diffusion coefficient; MK, mean kurtosis; MVI, microvascular invasion; AUC, area under the curve; PLR, positive likelihood ratio; NLR, negative likelihood ratio; DOR, diagnostic odds ratio; HCC, hepatocellular carcinoma.

### Subgroup Analysis of the Mean Apparent Diffusion Coefficient Value for Preoperative Diagnosis of Microvascular Invasion-Positive and Poorly Differentiated Hepatocellular Carcinoma

Due to differences in the study design, the number of included samples, and the examination equipment, clinical and methodological heterogeneity was inevitable. The results of the subgroup analysis are listed in **Table 3**. Interestingly, after grouping by subgroup (study design, sample size, machine type, number of b value, and maximum b value), the heterogeneity of the sensitivity and specificity decreased to varying degrees, suggesting that the subgroup might have been the source of heterogeneity. In addition, after grouping by maximum b value (≤800) and sample size (≤90), the AUC of the ADCmean for the diagnosis of pdHCC increased from 0.86 to 0.93, and the AUC of the MVI+ HCC increased from 0.78 to 0.81. Overall, each subgroup analysis had a good prediction effect.

**Table 3:** Subgroup analysis of the ADCmean value for diagnosis of MVI+ and poorly differentiated HCC.

Indicators/Subgroup	Groups (Studies)	AUC (95%CI)	Sensitivity (95% CI)	Specificity (95% CI)	PLR (95% CI)	NLR (95% CI)	DOR (95% CI)	I^2^	
								Sensitivity (%)	Specificity (%)
**Poorly differentiated HCC**									
**Study design**	Retrospective (n = 13)	0.87 (0.84, 0.90)	0.84 (0.76, 0.89)	0.75 (0.64, 0.84)	3.3 (2.2, 5.1)	0.22 (0.14, 0.33)	15 (7, 32)	57.32	85.14
	Prospective (n = 3)	0.84 (0.80, 0.87)	0.81 (0.61, 0.92)	0.78 (0.71, 0.84)	3.7 (2.8, 4.9)	0.24 (0.12, 0.51)	15 (7, 35)	75.69	28.46
**Sample size**	>90 (n = 7)	0.88 (0.85, 0.90)	0.86 (0.78, 0.91)	0.73 (0.62, 0.82)	3.2 (2.3, 4.5)	0.19 (0.13, 0.29)	16 (10, 27)	57.57	86.91
	≤90 (n = 9)	0.85 (0.82, 0.88)	0.78 (0.65, 0.88)	0.79 (0.67, 0.87)	3.7 (2.1, 6.5)	0.28 (0.15, 0.50)	13 (5, 39)	63.23	73.56
**Machine type**	3.0T (n = 7)	0.82 (0.78, 0.85)	0.81 (0.71, 0.88)	0.73 (0.66, 0.79)	3.0 (2.5, 3.6)	0.26 (0.17, 0.39)	12 (7, 18)	74.27	59.89
	1.5T (n = 9)	0.88 (0.85, 0.91)	0.84 (0.74, 0.91)	0.79 (0.62, 0.90)	4.0 (2.0, 8.0)	0.20 (0.11, 0.37)	20 (6, 63)	54.30	89.78
**Number of b value**	>3 (n = 7)	0.86 (0.83, 0.89)	0.78 (0.67, 0.86)	0.80 (0.70, 0.87)	3.9 (2.3, 6.4)	0.27 (0.16, 0.46)	14 (5, 37)	62.24	70.21
	≤3 (n = 9)	0.87 (0.84, 0.90)	0.87 (0.78, 0.93)	0.71 (0.58, 0.81)	3.0 (2.1, 4.3)	0.18 (0.10, 0.31)	17 (9, 32)	61.7	86.33
**Maximum b value**	>800 (n = 9)	0.81 (0.78, 0.85)	0.76 (0.68, 0.83)	0.73 (0.64, 0.81)	2.8 (2.2, 3.7)	0.32 (0.25, 0.42)	9 (6, 13)	34.64	79.08
	≤800 (n = 7)	0.93 (0.90, 0.95)	0.91 (0.78, 0.97)	0.80 (0.63, 0.90)	4.5 (2.2, 9.1)	0.11 (0.04, 0.30)	40 (10, 169)	78.23	87.92
**MVI(+) vs. MVI(-)**									
**Study design**	Retrospective (n = 5)	0.78 (0.74, 0.81)	0.73 (0.65, 0.80)	0.72 (0.57, 0.83)	2.6 (1.7, 3.9)	0.38 (0.30, 0.48)	7 (4, 12)	34.54	85.96
	Prospective (n = 3)	0.77 (0.74, 0.81)	0.74 (0.64, 0.82)	0.71 (0.59, 0.81)	2.6 (1.7, 4.0)	0.37 (0.25, 0.54)	7 (3, 15)	0	36.7
**Sample size**	>90 (n = 4)	0.76 (0.72, 0.80)	0.74 (0.67, 0.81)	0.63 (0.52, 0.73)	2.0 (1.6, 2.6)	0.40 (0.32, 0.51)	5 (3, 8)	25.48	79.83
	≤90 (n = 4)	0.81 (0.78, 0.85)	0.74 (0.64, 0.81)	0.80 (0.72, 0.87)	3.8 (2.6, 5.5)	0.33 (0.24, 0.46)	11 (6, 21)	0	36.13
**Machine type**	3.0T (n = 5)	0.77 (0.73, 0.80)	0.73 (0.65, 0.80)	0.73 (0.60, 0.83)	2.7 (1.8, 4.0)	0.37 (0.28, 0.49)	7 (4, 13)	0	70.66
	1.5T (n = 3)	0.78 (0.74, 0.81)	0.74 (0.65, 0.81)	0.70 (0.52, 0.83)	2.4 (1.5, 3.9)	0.37 (0.28, 0.49)	7 (3, 12)	20.9	84.49
**Number of b value**	>3 (n = 4)	0.73 (0.69, 0.77)	0.72 (0.63, 0.79)	0.76 (0.63, 0.86)	3.0 (1.9, 4.9)	0.37 (0.27, 0.51)	8 (4, 17)	0	68.92
	≤3 (n = 4)	0.78 (0.74, 0.81)	0.75 (0.68, 0.81)	0.67 (0.53, 0.78)	2.3 (1.6, 3.2)	0.37 (0.29, 0.47)	6 (4, 10)	12.73	84.8
**Maximum b value**	>800 (n = 5)	0.74 (0.70, 0.78)	0.73 (0.65, 0.79)	0.77 (0.67, 0.84)	3.1 (2.1, 4.6)	0.36 (0.27, 0.48)	9 (5, 16)	0	61.19
	≤800 (n = 3)	0.77 (0.73, 0.80)	0.75 (0.67, 0.82)	0.63 (0.47, 0.76)	2.0 (1.4, 2.9)	0.39 (0.30, 0.51)	5 (3, 9)	3.42	78.99

ADCmean, mean apparent diffusion coefficient; MVI, microvascular invasion; AUC, area under the curve; PLR, positive likelihood ratio; NLR, negative likelihood ratio; DOR, diagnostic odds ratio; HCC, hepatocellular carcinoma.

## Discussion

Hepatectomy and liver transplantation are currently the preferred treatment methods for HCC. Due to the invasive nature of surgery and the limited availability of organ transplantation, it is extremely important to determine the possibility of postoperative recovery and recurrence rate in patients before surgery. The HCC pathological grade and MVI are independent risk factors for recurrence and metastasis after hepatectomy or liver transplantation (56, 57). Therefore, preoperative prediction of pathological grade or MVI in HCC is crucial. The DKI is based on the non-Gaussian distribution model, which can better and more accurately reflect the subtle changes of tissue microstructure (58). IVIM adopts a multi-b-value scan and double exponential model fitting, which can more accurately reflect the diffusion of water molecules in tissues and microvascular blood perfusion, thereby better reflecting the heterogeneity of tumors (59). However, there are controversies as to whether the parameters of DKI and IVIM-DWI can be employed in the preoperative distinguishing of pathological grades and MVI in individuals with HCC. Therefore, 42 original studies were strictly included in this analysis to expand the sample size, and they were objectively and comprehensively evaluated to determine the diagnostic value of the DKI and IVIM-DWI parameters.

Based on SMDs, we showed that there were significant differences in the MK, MD, D, ADCmean, and ADCmin for preoperative prediction of the pathological grade or MVI in individuals with HCC. The D, ADCmean, and ADCmin positively correlated with the degree of differentiation of HCC. However, these findings are inconsistent with the conclusion of the meta-analysis by Surov et al. (60) that the ADCmean could not predict pathological grade and MVI in HCC. The reason may be that we included new studies (33–35, 38, 49, 50) and expanded the sample size. Moreover, various combination methods contributed to the differences. Surov et al. (60) combined the means of grades 1, 2, and 3 and MVI+/- and then compared whether there was an overlap between the combined means. In contrast, the SMDs were used as the effective index to distinguish well-, moderately, and poorly differentiated HCC and MVI+/- in our study. Similarly, the MK and MD could be used for preoperative distinguishing between pdHCC and non-pdHCC and between MVI+ and MVI-, with significant differences. The SMDs and 95% CIs were significantly away from the 0 reference line, which suggested that the MK and MD values were of great value in the identification of grades/MVI in HCC. The MK and MD values were the most representative parameters in DKI, which were able to reflect the complexity of tumor tissue microstructure and had potential correlation with tumor invasive biological behavior (38). Compared with non-pdHCC, pdHCC had greater heteromorphism, and the proliferation capacity of cancer tissues was more vigorous, which led to complex tissue structure and non-Gaussian distribution of the water molecule movement, thereby resulting in a higher MK value and a lower MD value.

Interestingly, some studies (22, 25, 34) have suggested that the D* or f values could predict HCC pathological grades, while other studies (18, 35, 39) did not confirm such conclusions. Our study suggested that there was no significant benefit of D* or f values in predicting HCC pathological grades. The reason may be that the D* value is mainly related to microcirculation blood flow velocity; thus, this can lead to inaccurate measurements under subjective dynamics. In addition, the D* value could not truthfully reflect the real value of cancer focus because the D* value is easily affected by the changes of machine signal and noise. Similarly, the f value indicates the microcirculation perfusion fraction, and the repeatability of measurement is poor because the microcirculation blood flow is dynamic at all times.

Importantly, our study suggested that the MK, D, ADCmean, and ADCmin had a higher diagnostic efficacy to predict pdHCC. Compared with the ADCmean and ADCmin, the D value had higher sensitivity, specificity, and AUC. Similarly, the AUC of the pdHCC predicted by the MK value was 0.89, which was higher than that predicted by the ADCmean and ADCmin, and the specificity was as high as 94%. The reason might be that the MK value is based on a non-Gaussian model; thus, it could reflect the diffusion characteristics of water molecules *in vivo* as a whole and could more truly reflect the movement state of water molecules in the lesion. Compared with the meta-analysis of Yang et al. (52), our study latest suggested that the D value had excellent diagnostic efficacy in predicting wdHCC, with a sensitivity of 87%, specificity of 83%, and AUC of 0.92; moreover, our study subdivided the ADC value into the mean and minimum ADC value on the basis of expanding the sample size, thereby making the combined results more reliable.

Furthermore, compared with the ADCmean, our study suggested that the D value had higher sensitivity (80%) and specificity (80%) in predicting MVI+ HCC, and the summary AUC of the D value was significantly higher than that of the ADCmean (*Z* = −2.208, *P* = 0.027), indicating that the D value was better and more sensitive in predicting MVI+ HCC. The reason might be that the ADC value ignores the influence of microcirculation perfusion in the cancer focus; thus, the D value is more realistic than the ADC value, given that the D value distinguishes the diffusion of pure water molecules and microcirculation perfusion in the tissue by changes in the b value (61).

Our study comprehensively and systematically evaluated the power of the DKI, IVIM, and DWI parameters for preoperative prediction of the pathological grade and MVI in HCC. The quality of the included studies was acceptable, and there was no publication bias in the studies according to Egger’s or Begg’s test. Moreover, we performed the subgroup analysis of the ADCmean value for the diagnosis of MVI+ HCC and pdHCC. Interestingly, after grouping by maximum b value (≤800) and sample size (≤90), the AUC of the ADCmean for the diagnosis of pdHCC increased from 0.86 to 0.93, and the AUC of the MVI+ HCC increased from 0.78 to 0.81. Overall, each subgroup analysis had a good prediction effect.

However, our study had some limitations. First, most studies were retrospective studies, which increased the risk of confusion bias to a certain extent. Second, the sample size of the MK, MD, D*, and f values was not large enough. Therefore, further studies with a larger sample size and of prospective nature are needed to prove our results. Finally, most studies were conducted in Asia, which introduced a certain regional bias.

## Conclusion

Our meta-analysis showed that the DKI parameters (MD and MK) and the IVIM-DWI parameters (D value, ADCmean, and ADCmin) can be used as a noninvasive and simple preoperative examination method to predict the pathological grade and MVI in HCC. Compared with the ADCmean and ADCmin, the MD and D values showed a higher diagnostic efficacy in predicting the grades of HCC, and the D value had superior diagnostic efficacy to the ADCmean in predicting MVI+ in HCC. However, f values cannot be used as an effective parameter to predict the grades and MVI in HCC. It is quite helpful when making a clinical treatment plan, preoperative prognosis evaluation, and follow-up research.

## Data Availability Statement

All datasets generated for this study are included in the article/**Supplementary Material**.

## Author Contributions

Research route design: FW, CYY, and DZ. Draft of the article: FW. Data acquisition: CHW, CYY, and FW. Data analysis: FW and CYY. Review and editing of the article: DZ and YY. All authors contributed substantially to the preparation of the article.

## Funding

This work was supported by Talent project of Chongqing (Dong Zhang, CQYC202103075).

## Conflict of Interest

The authors declare that the research was conducted in the absence of any commercial or financial relationships that could be construed as a potential conflict of interest.

## Publisher’s Note

All claims expressed in this article are solely those of the authors and do not necessarily represent those of their affiliated organizations, or those of the publisher, the editors and the reviewers. Any product that may be evaluated in this article, or claim that may be made by its manufacturer, is not guaranteed or endorsed by the publisher.
