# Supplementary material for: The Roles of Diffusion Kurtosis Imaging and Intravoxel Incoherent Motion Diffusion-Weighted Imaging Parameters in Preoperative Evaluation of Pathological Grades and Microvascular Invasion in Hepatocellular Carcinoma
Source: Front Oncol. 2022 May 11;12:884854. doi: 10.3389/fonc.2022.884854 (PMC9131658; doi:10.3389/fonc.2022.884854)
Supplement: Supplementary file 1 [file DataSheet_1.doc]

Supplementary Material

# 1.Supplementary Table

**Supplementary Table S1** some parameters of diagnostic studies

| **Indicators** | **study** | **year** | **Sensitivity(%)** | **Specificity(%)** | **AUC** | **TP** | **FP** | **FN** | **TN** |
| --- | --- | --- | --- | --- | --- | --- | --- | --- | --- |
| **poorly-differentiated HCC** |  |  |  |  |  |  |  |  |  |
| MK | Cao et al(38) | 2019 | 72.4 | 82.2 | 0.81 | 33 | 5 | 12 | 24 |
|  | Wu et al(34) | 2020 | 53.06 | 97.44 | 0.765 | 26 | 1 | 23 | 38 |
|  | Wang et al(32) | 2020 | 78.26 | 94.92 | 0.93 | 46 | 4 | 13 | 65 |
| D | Granata et al(22) | 2016 | 100 | 74 | 0.947 | 18 | 11 | 0 | 33 |
|  | Woo et al(18) | 2014 | 88.9 | 79.2 | 0.838 | 16 | 5 | 2 | 19 |
|  | Wei et al(39) | 2019 | 94.29 | 91.07 | 0.969 | 33 | 5 | 2 | 51 |
|  | Wei et al(39) | 2019 | 94.29 | 80.36 | 0.873 | 33 | 11 | 2 | 45 |
|  | Wei et al(39) | 2019 | 91.43 | 91.07 | 0.938 | 32 | 5 | 3 | 51 |
|  | Wu et al(34) | 2020 | 53.06 | 84.62 | 0.757 | 21 | 8 | 18 | 41 |
|  | Shan et al(25) | 2017 | 84 | 56 | 0.726 | 21 | 37 | 4 | 47 |
|  | Sokmen et al(31) | 2019 | 63.6 | 66.7 | 0.833 | 9 | 9 | 5 | 19 |
|  | Shi et al(32) | 2020 | 78.1 | 83 | 0.811 | 21 | 4 | 6 | 21 |
| ADCmean | Heo et al(11) | 2010 | 78 | 83 | Na | 7 | 3 | 2 | 15 |
|  | Nishie et al(12) | 2011 | 73.1 | 72.9 | Na | 19 | 16 | 7 | 43 |
|  | Guo et al(19) | 2015 | 100 | 54.5 | Na | 11 | 7 | 0 | 9 |
|  | Li et al(24) | 2016 | 69.57 | 73.39 | 0.74 | 16 | 58 | 7 | 160 |
|  | Wang et al(32) | 2020 | 94.2 | 57.63 | 0.815 | 65 | 25 | 4 | 34 |
|  | Woo et al(18) | 2014 | 72.2 | 75 | 0.728 | 13 | 6 | 5 | 18 |
|  | Granata et al(22) | 2016 | 100 | 100 | 1 | 18 | 0 | 0 | 44 |
|  | Jiang et al(26) | 2017 | 96.55 | 73.33 | 0.906 | 28 | 60 | 1 | 165 |
|  | Wei et al(39) | 2019 | 85.71 | 80.36 | 0.861 | 30 | 11 | 5 | 45 |
|  | Wei et al(39) | 2019 | 71.43 | 82.14 | 0.806 | 25 | 10 | 10 | 46 |
|  | Wei et al(39) | 2019 | 82.86 | 78.57 | 0.84 | 29 | 12 | 6 | 44 |
|  | Shan et al(25) | 2017 | 76 | 73.8 | 0.787 | 19 | 22 | 6 | 62 |
|  | Sokmen et al(31) | 2019 | 71.4 | 67.7 | 0.857 | 10 | 9 | 4 | 19 |
|  | Wu et al(34) | 2020 | 53.06 | 82.05 | 0.713 | 21 | 9 | 18 | 40 |
|  | Chang et al(16) | 2014 | 83.7 | 89.8 | 0.89 | 36 | 10 | 7 | 88 |
|  | Cao et al(38) | 2019 | 75.9 | 60 | 0.74 | 22 | 18 | 7 | 27 |
|  | Muhi et al(10) | 2009 | 92.86 | 26.47 | Na | 13 | 24 | 1 | 9 |
|  | Iwasa et al(21) | 2016 | 71 | 82 | 0.79 | 7 | 6 | 3 | 26 |
| ADCmin | Nakanishi et al(13) | 2012 | 78 | 81 | Na | 14 | 6 | 4 | 26 |
|  | Li et al(24) | 2016 | 78.26 | 61.47 | 0.73 | 18 | 84 | 5 | 134 |
|  | Moriya et al(26) | 2017 | 100 | 54 | Na | 9 | 22 | 0 | 25 |
| **well-differentiated HCC** |  |  |  |  |  |  |  |  |  |
| D | Zhu et al(30) | 2018 | 92.9 | 81.3 | 0.909 | 13 | 9 | 1 | 39 |
|  | Zhu et al(30) | 2018 | 92.9 | 87.5 | 0.911 | 13 | 6 | 1 | 42 |
|  | Zhou et al(35) | 2021 | 86.7 | 78.2 | 0.841 | 13 | 12 | 2 | 43 |
|  | Shan et al(25) | 2017 | 77.8 | 84.6 | 0.84 | 14 | 14 | 4 | 77 |
| ADCmean | Zhu et al(30) | 2018 | 78.6 | 83.3 | 0.843 | 11 | 8 | 3 | 40 |
|  | Zhu et al(30) | 2018 | 85.7 | 79.2 | 0.852 | 12 | 10 | 2 | 38 |
|  | Zhou et al(35) | 2021 | 73.3 | 85.5 | 0.821 | 11 | 8 | 4 | 47 |
|  | Shan et al(25) | 2017 | 94.4 | 85.7 | 0.903 | 17 | 13 | 1 | 78 |
|  | Chang et al(16) | 2014 | 82.4 | 83.2 | 0.88 | 28 | 18 | 6 | 89 |
|  | Jiang et al(26) | 2017 | 94.88 | 100 | 0.986 | 37 | 0 | 2 | 215 |
|  | Sandrasegaran et al(12) | 2013 | 85 | 86 | 0.87 | 23 | 2 | 4 | 12 |
|  | Shankar et al(23) | 2016 | 75 | 50 | Na | 6 | 6 | 2 | 6 |
|  | Muhi et al(10) | 2009 | 63.16 | 91.67 | Na | 25 | 4 | 14 | 43 |
| **MVI(+) vs MVI(-)** |  |  |  |  |  |  |  |  |  |
| D | Wei et al(51) | 2019 | 78.2 | 75 | 0.815 | 43 | 20 | 12 | 60 |
|  | Li et al(46) | 2018 | 81 | 85 | 0.874 | 17 | 3 | 4 | 17 |
|  | Zhao et al(47) | 2018 | 66.7 | 88.9 | 0.753 | 12 | 4 | 6 | 29 |
|  | Chen et al(49) | 2021 | 90 | 78.79 | 0.912 | 27 | 7 | 3 | 26 |
| ADCmean | Zhao et al(45) | 2017 | 79.15 | 50.47 | 0.69 | 167 | 53 | 44 | 54 |
|  | Wei et al(51) | 2019 | 70.9 | 65 | 0.746 | 39 | 28 | 16 | 52 |
|  | Okamura et al(42) | 2016 | 75.8 | 77.5 | 0.772 | 25 | 9 | 8 | 33 |
|  | Li et al(46) | 2018 | 76.2 | 65 | 0.72 | 16 | 7 | 5 | 13 |
|  | Zhao et al(47) | 2018 | 60.6 | 88.9 | 0.67 | 11 | 4 | 7 | 29 |
|  | Xu et al(41) | 2014 | 66.7 | 78.6 | 0.711 | 26 | 15 | 13 | 55 |
|  | Chen et al(49) | 2021 | 76.67 | 84.85 | 0.831 | 23 | 5 | 7 | 28 |
|  | Wang et al(50) | 2021 | 78.95 | 57.14 | 0.6805 | 34 | 24 | 9 | 33 |

*ADCmean,mean Apparent diffusion coefficient; ADCmin,minimum Apparent diffusion coefficient; D,tissue Diffusivity; MVI,Microvascular invasion; AUC,Area under the curve; TP,True positive; TN,True negative; FP,False positive; FN,False negative; HCC,hepatocellular carcinoma.*

2. **Supplementary Figures**

**Supplementary Figure S1A.** Sensitivity analysis chart of ADCmean distinguished MVI(-) versus MVI(+), and the result of sensitivity analysis was stable and reliable.

**Supplementary Figure S1B.** Sensitivity analysis chart of ADCmin distinguished MVI(-) versus MVI(+), but the study of Kim et al affected the stability of the results.

**Supplementary Figure S1C.** Sensitivity analysis chart of D values distinguished MVI(-) versus MVI(+), and the result of sensitivity analysis was stable and reliable.

**Supplementary Figure S1D.** Sensitivity analysis chart of D* values distinguished MVI(-) versus MVI(+), and the result of sensitivity analysis was stable and reliable.

**Supplementary Figure S1E.** Sensitivity analysis chart of f values distinguished MVI(-) versus MVI(+), and the result of sensitivity analysis was stable and reliable.

**Supplementary Figure S1F.** Sensitivity analysis chart of MD values distinguished MVI(-) versus MVI(+), and the result of sensitivity analysis was stable and reliable.

**Supplementary Figure S1G.** Sensitivity analysis chart of MK values distinguished MVI(-) versus MVI(+), and the result of sensitivity analysis was stable and reliable.

**Supplementary Figure S2.** Forest plot of the ADCmin between MVI(-) and MVI(+). After removing the study of Kim et al, the SMD indicated that MVI(+) had a significantly lower ADCmin than MVI(-). MVI, microvascular invasion; SMD, standardized mean difference.

A:Sensitivity analysis chart of ADCmean distinguished wdHCC versus mdHCC

B:Sensitivity analysis chart of ADCmean distinguished wdHCC versus pdHCC

C:Sensitivity analysis chart of ADCmean distinguished mdHCC versus pdHCC

**Supplementary Figure S3A-C.** Sensitivity analysis chart of ADCmean distinguished grades, and the results of sensitivity analysis were stable and reliable except ADCmean value to identify wdHCC versus mdHCC. The study of Jiang et al affected the stability of the results.

A:Sensitivity analysis chart of ADCmin distinguished wdHCC versus mdHCC

B:Sensitivity analysis chart of ADCmin distinguished wdHCC versus pdHCC

C:Sensitivity analysis chart of ADCmin distinguished mdHCC versus pdHCC

**Supplementary Figure S4A-C.** Sensitivity analysis chart of ADCmin distinguished grades, and the results of sensitivity analysis were stable and reliable.

A:Sensitivity analysis chart of D values distinguished wdHCC versus mdHCC

B:Sensitivity analysis chart of D values distinguished wdHCC versus pdHCC

C:Sensitivity analysis chart of D values distinguished mdHCC versus pdHCC

**Supplementary Figure S5A-C.** Sensitivity analysis chart of D values distinguished grades, and the result of sensitivity analysis was stable and reliable except to identify wdHCC versus pdHCC and mdHCC versus pdHCC. The study of Shan et al and Granata et al affected the stability of the results.

A:Sensitivity analysis chart of D* values distinguished wdHCC versus mdHCC

B:Sensitivity analysis chart of D* valuesdistinguished wdHCC versus pdHCC

C:Sensitivity analysis chart of D* values distinguished mdHCC versus pdHCC

**Supplementary Figure S6A-C.** Sensitivity analysis chart of D* values distinguished grades, and the results of sensitivity analysis were stable and reliable.

A:Sensitivity analysis chart of f values distinguished wdHCC versus mdHCC

B:Sensitivity analysis chart of f values distinguished wdHCC versus pdHCC

C:Sensitivity analysis chart of f values distinguished mdHCC versus pdHCC

**Supplementary Figure S7A-C.** Sensitivity analysis chart of f values distinguished grades, and the results of sensitivity analysis were stable and reliable.

**Supplementary Figure S8.** Forest plot of the ADCmean between wdHCC and mdHCC. After removing the study of Jiang et al, the SMD indicated that mdHCC had a significantly lower ADCmean than wdHCC. wd-HCC, well differentiated hepatocellular carcinoma; md-HCC, moderately differentiated hepatocellular carcinoma; SMD, standardized mean difference.

**Supplementary Figure S9.** Forest plot of the D values between wdHCC and pdHCC. After removing the study of Shan et al and Granata et al, the SMD indicated that pdHCC had a significantly lower D values than wdHCC. wd-HCC, well differentiated hepatocellular carcinoma; pd-HCC, poorly differentiated hepatocellular carcinoma; SMD, standardized mean difference.

**Supplementary Figure S10.** Forest plot of the D values between mdHCC and pdHCC. After removing the study of Shan et al and Granata et al, the SMD indicated that pdHCC had a significantly lower D values than mdHCC. md-HCC, moderately differentiated hepatocellular carcinoma; pd-HCC, poorly differentiated hepatocellular carcinoma; SMD, standardized mean difference.
